# A preliminary study of the concentration of metallic elements in the blood of patients with multiple sclerosis as measured by ICP-MS

**DOI:** 10.1038/s41598-020-69979-9

**Published:** 2020-08-04

**Authors:** Marcela de Oliveira, Thiago Marcelo Ribeiro Gianeti, Fernando Coronetti Gomes da Rocha, Paulo Noronha Lisboa-Filho, Marina Piacenti-Silva

**Affiliations:** 1 Department of Physics, School of Sciences, São Paulo State University, Av. Eng. Luiz Edmundo Carrijo Coube, 14-01-Vargem Limpa, Bauru, SP 17033-360 Brazil; 2 Ourofino Agrociência, Ribeirão Preto, SP 14026-020 Brazil; 3 Department of Neurology, Psychology and Psychiatry, Medical School, São Paulo State University, Botucatu, SP 18618-687 Brazil

**Keywords:** Multiple sclerosis, Mass spectrometry, Environmental impact, Risk factors

## Abstract

It is estimated that multiple sclerosis (MS) affects 35,000 Brazilians and 2.5 million individuals worldwide. Many studies have suggested a possible role of metallic elements in the etiology of MS, but their concentration in the blood of MS patients is nonetheless little investigated in Brazil. In this work, these elements were studied through Inductively Coupled Plasma Mass Spectrometry (ICP-MS), whose analysis provides a tool to quantify the concentrations of metal elements in the blood samples of individuals with neurodegenerative disorders. This study aimed to compare the concentration of metallic elements in blood samples from patients with MS and healthy individuals. Blood was collected from 30 patients with multiple sclerosis and compared with the control group. Blood samples were digested in closed vessels using a microwave and ICP-MS was used to determine the concentrations of 12 metallic elements (Ba, Be, Ca, Co, Cr, Cu, Fe, Mg, Mo, Ni, Pb and Zn). In MS patients, we observed a reduction in the concentrations of beryllium, copper, chromium, cobalt, nickel, magnesium and iron. The mean concentration of lead in blood was significantly elevated in the MS group. However, no difference was observed in the concentrations of Mo, Ba, Ca and Zn in blood samples from MS patients and the control group. According to our data, there is a possible role for the concentrations of 8 of the 12 evaluated metallic elements in multiple sclerosis. Abnormalities in transition metals levels in biological matrices have been reported in several neurological diseases.

## Introduction

Multiple Sclerosis (MS) is a neuroinflammatory disease with an etiology that may involve genetic and environmental factors^1^. MS affects over 400,000 people in the United States, 35,000 Brazilians and 2.5 million worldwide, making it a pressing issue in healthcare^2^. It is recognized worldwide, however, that reported incidence rates and prevalence vary considerably between regions and populations, indicating that environmental and climatic factors can possibly also contribute towards etiology^3,4^. Groups that have been identified as the highest risk for the development of MS, are those involved in paper manufacturing, leather, wood processing, welding, metal, printing, electronics, textiles and agriculture^5^. Environmental agents are believed to trigger an inflammatory process and, consequently, an autoimmune process to myelin proteins in individuals with a genetic predisposition, with a consequent loss of neurological function^6,7^. Exposure and the subsequent absorption of toxic materials such as metallic nanoparticles (NPs) and metallic dioxides onto human membranes have been reported as environmental agents and, as such, contribute to oxidative stress and the pathogenesis of neurological diseases^8–10^. Therefore, examining the concentration of the metallic NPs as environmental agents can help to explore one of the possible causes of MS.

The investigation into metallic elements in previous studies has been based on experimental disease models, post-mortem materials, in vitro conditions and in vivo tissue (hair or blood cells), all of which have pointed to the accumulation or depletion of trace elements in the body^9^. However, there is still great controversy, because these studies have considered a small number of elements and are hampered by methodological limitations in terms of analytical quality, such as a reduced number of samples and the different techniques applied^11^. There is a lack of information concerning exposure to heavy metals as potential etiologic factor for MS, and their concentrations in blood of MS patients have been little investigated in Brazil. Therefore, the aim of this study was to compare the concentration of metals by ICP-MS, in the blood of patients with multiple sclerosis and healthy controls, in order to investigate the role of these metal elements as environmental cofactors for multiple sclerosis.

## Materials and methods

### Sampling

The multiple sclerosis group included 30 patients aged 25–63 (23 women and 7 men) with definite MS, diagnosed according to the McDonald criteria^12^^,^ who were recruited from the Hospital of Clinics—Botucatu Medical School (HC-FMB). The control group consisted of 30 healthy volunteers aged 22–57 (15 women and 15 men) living in the same urban area. Both groups had normal diet, without dietary restrictions. The subjects were selected with the following exclusion factors: heart, kidney, liver or respiratory diseases; active infections; abnormalities of intestinal absorption; thyroid hormone therapy; vitamin or mineral supplement intake; lithium therapy; vegetarian diet; artificial metallic bodies; neurological diseases other than MS. The research ethics committee of Hospital of Clinics—Botucatu Medical School (CEP-HC-FMB) approved the study and all procedures performed involving human participants were in accordance with the relevant guidelines and regulations. All the participants gave written informed consent to participate in the study, in accordance with the CEP-HC-FMB. The informed consent was obtained from all the subjects (both healthy controls and patients) to participate in the study.

After completing the selection, 10 mL blood samples were taken from all subjects in accordance with standard procedures and collected in polystyrene tubes, with all sterilized materials and absence of anticoagulant. All samples were freeze-dried for a period of 24 h at − 60 °C and a pressure of − 780 mmHg (Alpha 1–2 LDplus Freeze Drier) and stored deep-frozen until analysis. In a vessel, 3 mL of HNO_3_ and 2 mL of H_2_O_2_ were added to 0.2 g of blood, freeze-dried and then microwave assisted digestion was carried out in closed vessels in an Milestone ETHOSEASY microwave (MW) oven with MAX-44 rotor. The following steps for MW digestion were applied: (i) 15 min of temperature ramp up to 180 °C and (ii) 15 min at 180 °C. After cooling down to room temperature, the digested samples were filled up to 25 mL using deionized water (following dilution 1.2 + 25 (v + v)), and stored at 4 °C until analysis by ICP-MS. The reference blood sample was digested under the same conditions as the samples, in addition to the blank samples. Nitric acid and hydrogen peroxide were considered as blank of the analytical method. All glassware and instruments used were properly washed and maximum care was taken to avoid trace element contamination of the samples^13,14^.

### Analytical measurements

#### Inductively coupled plasma mass spectrometry

Inductively coupled plasma mass spectrometry (ICP-MS), with its multi-element determination capability, wide dynamic range and high sensibility, which is used for the quantification of several, possibly pathological, elements in body fluids^15^^,^ was used in this study. From digested samples, the concentrations of the metallic elements Barium (Ba), Beryllium (Be), Calcium (Ca), Cobalt (Co), Chromium (Cr), Copper (Cu), Iron (Fe), Magnesium (Mg), Molybdenum (Mo), Nickel (Ni), Lead (Pb) and Zinc (Zn) were quantified by ICP-MS (NexION 300D Model, PerkinElmer). To quantify the concentrations of elements in blood, the following dilution 1.2 + 25 (v + v) was used.

The calibration curves were performed with multielement solutions from Specsol Brand Standards (traceable by National Institute of Standards and Technology—NIST). The accuracy of the method was carried out through the analysis of the Seronorm Trace Elements Whole Blood L-1, L-2 & L-3 (Lot 1702825—SERO). Seronorm Trace Elements Whole Blood is an accuracy control for the analyses of trace elements and heavy metals in human whole blood, and was used as reference materials.

Differences between the reference and experimentally determined values obtained for the concentrations of metallic elements present in the analyzed references were less than 20% for all references, ensuring the correct quantification of the analysis by ICP-MS.

### Statistical analysis

Statistical analyses were performed in SPSS Statistics software. We reported the means and standard deviations of normally distributed values. Independent sample *t*-tests were performed to compare the concentrations of metallic elements in blood from patients and the control group, and statistical analysis was carried out with a Mann Whitney test, with *p* < 0.05, when the variables were not normally distributed.

### Ethics approval

The research ethics committee of Hospital of Clinics—Botucatu Medical School (CEP – HC-FMB) approved the study and all procedures performed involving human participants were in accordance with the relevant guidelines and regulations (SIPE:147/2017). The study was registered on the National Research Platform (Plataforma Brasil—CAAE: 80261717.5.00005411).

### Consent to participate

The informed consent was obtained from all the subjects (both healthy controls and patients) to participate in the study.

## Results and discussion

Evidence on the possible relationship between multiple sclerosis and metallic elements mainly arise from case–control studies in which metal concentrations have been evaluated in different biological specimens such as blood, serum, hair, urine and cerebrospinal fluid^16^. The results obtained in this work and in several studies investigating metallic elements in MS patients may be observed in Table 1. We observed that our values in whole blood samples are higher than the results obtained in other studies that analyzed serum and cerebrospinal fluid. Forte et al.^17^ analyzed the same type of sample as our study, and in general the data coincide for whole blood. We can also observe that the type of preparation, analytical technique, and number of the samples are fundamental for interpretation of the results about metallic elements.

**Table 1:** Metallic element concentrations in patients with multiple sclerosis and control in different studies.

Element	References	Sample type	Technical analysis	Concentration (µg/L)		*p*
				CONTROL	MS	
Ba	This work	B	ICP-MS	5.05 ± 6.43	3.25 ± 2.45	0.897
	Forte et al.^17^	B	ICP-AES	1.17 ± 0.54	1.43 ± 0.11	NS
	Visconti et al.^19^	S	ICP-MS	0.69 ± 0.42	1.32 ± 0.54	< 0.05*
	Alimonti et al.^9^	S	ICP-MS	0.59 (0.43–0.92)	0.61 (0.44–0.81)	NS
	Janghorbani et al.^13^	P	ICP-AES	1.01 ± 0.01	1.01 ± 0.01	NS
	This work	B	ICP-MS	0.22 ± 0.013	0.0003 ± 0.002	0.000*
Be	Visconti et al.^19^	S	ICP-MS	0.26 ± 0.11	0.23 ± 0.06	NS
	Alimonti et al.^9^	S	ICP-MS	0.21 (0.31–0.32)	0.34 (0.21–0.45)	< 0.0003*
Ca	This work	B	ICP-MS	77,029.43 ± 53,013.06	55,716.59 ± 18,393.67	0.439
	Forte et al.^17^	B	ICP-AES	68,006 ± 10,517	65,410 ± 11,264	< 0.05*
	Visconti et al.^19^	S	ICP-AES	65,310 ± 5,620	86,017 ± 11,115	< 0.05*
	Alimonti et al.^9^	S	ICP-AES	64,236 (59,380–68,340)	73,039 (68,786–76,417)	< 0.0003*
	Janghorbani et al.^13^	P	ICP-AES	70,400 ± 420	58,300 ± 550	< 0.001*
Co	This work	B	ICP-MS	1.46 ± 0.79	0.0091 ± 0.0499	0.000*
	Visconti et al.^19^	S	ICP-MS	0.016 ± 0.09	0.21 ± 0.11	NS
	Alimonti et al.^9^	S	ICP-MS	0.16 (0.10–0.25)	0.14 (0.09–0.24)	NS
Cr	This work	B	ICP-MS	38.96 ± 148.07	6.03 ± 8.37	0.001*
	Visconti et al.^19^	S	ICP-MS	0.14 ± 0.05	0.36 ± 0.22	0.85
	Alimonti et al.^9^	S	ICP-MS	0.15 (0.12–0.21)	0.17 (0.13–0.23)	NS
	Janghorbani et al.^13^	P	ICP-AES	1.09 ± 0.02	1.17 ± 0.02	< 0.01*
Cu	This work	B	ICP-MS	1,052.29 ± 487.05	721.21 ± 222.78	0.000*
	Forte et al.^17^	B	ICP-AES	926 ± 144	1,445 ± 481	< 0.05*
	Melo et al.^21^	CF	ICP-MS	8.67 ± 0.49	10.90 ± 1.11	< 0.05*
	Visconti et al.^19^	S	ICP-AES	953 ± 75	1,034 ± 228	< 0.05*
	Alimonti et al.^9^	S	ICP-AES	951 (808–1,073)	940 (819–1,031)	NS
	Iranmanesh et al.^20^	S	CAA	110.37 ± 37.10	88.58 ± 19.56	< 0.05*
	Sedighi et al.^28^	S	AAS	88.9	93.7	0.46
	Janghorbani et al.^13^	P	ICP-AES	1.06 ± 0.02	1.67 ± 0.03	< 0.001*
Fe	This work	B	ICP-MS	496,556 ± 43,797	424,319 ± 51,525	0.000*
	Forte et al.^17^	B	ICP-AES	546,285 ± 69,705	482,748 ± 83,763	< 0.05*
	Visconti et al.^19^	S	ICP-AES	1686 ± 547	1,318 ± 527	< 0.10*
	Alimonti et al.^9^	S	ICP-AES	1,610 (1,228–1995)	920 (752–1,294)	< 0.0003*
	Iranmanesh et al.^20^	S	CAA	103.95 ± 33.81	127.04 ± 34.67	< 0.05*
	Janghorbani et al.^13^	P	ICP-AES	4.25 ± 0.02	4.13 ± 0.03	< 0.01*
Mg	This work	B	ICP-MS	36,416 ± 4,155.48	27,837 ± 4,127.48	0.000*
	Forte et al.^17^	B	ICP-AES	40,392 ± 5,657	36,784 ± 5,804	NS
	Alimonti et al.^9^	S	ICP-AES	17,424 (16,501–19,086)	18,391 (17,458–19,638)	NS
	Janghorbani et al.^13^	P	ICP-AES	12,330 ± 250	13,470 ± 330	< 0.1*
Mo	This work	B	ICP-MS	2.03 ± 3.22	1.46 ± 0.72	0.879
	Forte et al.^17^	B	ICP-MS	2.94 ± 1.65	2.11 ± 0.83	< 0.05*
	Visconti et al.^19^	S	ICP-MS	0.86 ± 0.35	1.45 ± 0.49	< 0.05*
	Alimonti et al.^9^	S	ICP-MS	0.84 (0.58–1.22)	1.14 (0.82–1.43)	NS
	Janghorbani et al.^13^	P	ICP-AES	0.08 ± 0.04	0.17 ± 0.06	NS
Ni	This work	B	ICP-MS	13.34 ± 37.04	1.92 ± 4.43	0.000*
	Visconti et al.^19^	S	ICP-MS	0.44 ± 0.35	1.62 ± 1.24	< 0.05*
	Alimonti et al.^9^	S	ICP-MS	0.39 (0.34–0.71)	0.81 (0.41–1.37)	< 0.0003*
	Janghorbani et al.^13^	P	ICP-AES	0.001 ± 0.009	0.016 ± 0.012	NS
Pb	This work	B	ICP-MS	12.46 ± 3.89	24.55 ± 19.94	0.000*
	Forte et al.^17^	B	ICP-MS	35.6 ± 20.1	24.3 ± 12.7	< 0.05*
	Visconti et al.^19^	S	ICP-MS	0.65 ± 0.34	0.53 ± 0.45	NS
	Alimonti et al.^9^	S	ICP-MS	0.53 (0.34–0.71)	0.40 (0.26–0.54)	NS
	Aliomrani et al.^36^	B	AAS	46.1 ± 22	44.7 ± 25	0.625
	Janghorbani et al.^13^	P	ICP-AES	0.94 ± 0.01	0.96 ± 0.02	NS
	Dehghanifiroozabadi et al.^22^	S	GFAAS	33.8 ± 28.8	75.6 ± 9.35	0.003*
Zn	This work	B	ICP-MS	4,298.35 ± 623.14	4,270.15 ± 823.56	0.883
	Forte et al.^17^	B	ICP-AES	6,579 ± 1,015	5,998 ± 1,492	< 0.05*
	Melo et al.^21^	CF	ICP-MS	23.5 ± 3.2	19.0 ± 1.9	NS
	Visconti et al.^19^	S	ICP-AES	781 ± 120	864 ± 160	NS
	Alimonti et al.^9^	S	ICP-AES	795 (703–897)	650 (591–710)	< 0.0003*
	Iranmanesh et al.^20^	S	CAA	14.1 ± 3.2	10.9 ± 2.1	< 0.05*
	Sedighi et al.^28^	S	AAS	36.7	40.9	0.25
	Janghorbani et al.^13^	P	ICP-AES	288.11 ± 2.01	235.20 ± 2.63	< 0.001*
	Pawlitzki et al.^18^	S	AAS	14.6 ± 2.3	12.5 ± 2.1	< 0.05*

Values are reported as means ± standard deviation, and median (interquartile ranges).

*B* Blood, *S* Serum, *CF* Cerebrospinal Fluid, *P* Plasma, *ICP-OES* Inductively Coupled Plasma Optical Emission Spectrometry, *ICP-AES* Inductively Coupled Plasma Atomic Emission Spectrometry, *ICP-MS* Inductively Coupled Plasma Mass Spectrometry, *AAS* Atomic Absorption Spectrophotometer, *CAA* Colorimetric Auto-Analyzer, *GFAAS* Graphite Furnace Atomic Absorption Spectrometry, *NS* non-significant statistical difference.

*Significant statistical difference.

We can note that the literature suggests a possible relationship between the imbalance of metal levels and multiple sclerosis. In our results, only the mean concentration of lead in whole blood was significantly elevated in the MS group. However, we observed a reduction in the concentrations of all other analyzed elements in MS patients. Beryllium, copper, chromium, cobalt, nickel, magnesium and iron had a statistically significant reduction, but there were no significant differences for the concentrations of Mo, Cs, Ba, Ca, Se and Zn in blood samples from patients and the control group.

We noted that all concentrations were lower for the patient group, except for Pb. Our results for Cu, Fe and Zn agree with those observed by Alimonti et al.^9^^,^ who showed a decreased concentration in the blood of MS patients. Pawlitzki et al.^18^ also found lower serum zinc levels in patients with multiple sclerosis. However, Janghorbani et al.^13^ and Visconti et al.^19^ found an increase in the levels of these metallic elements in the serum of MS subjects. Ours results showed that the concentrations of copper, magnesium, chromium and nickel also were lower in the MS group. Similar findings were found by Iranmanesh et al.^20^^,^ who noted that Cu levels decreased in the serum of MS subjects, and by Forte et al.^17^ who showed a relative Mg insufficiency in the same group. Forte et al.^17^ also observed that Mg concentrations were reduced in the cerebrospinal fluid (CSF) of 18 patients with multiple sclerosis.

Nevertheless, some studies have shown an increase in metal levels, where chromium and Ni concentrations were higher in the disease group^9,13,19^. The results observed by Melo et al^21^ and Janghorbani et al.^13^ showed an increase in Cu levels in the CSF and serum of MS patients, respectively. Additionally, Fe concentrations were increased in the cases evaluated by Iranmanesh et al.^20^^,^ who analyzed the serum of 25 patients with multiple sclerosis.

With respect to Pb, this was the only element that showed an increase in concentration levels for MS patients in our study, and this finding agrees with the most recent studies performed by Janghorbani et al.^13^ and Dehghanifiroozabadi et al.^22^.

**Figure 1:**
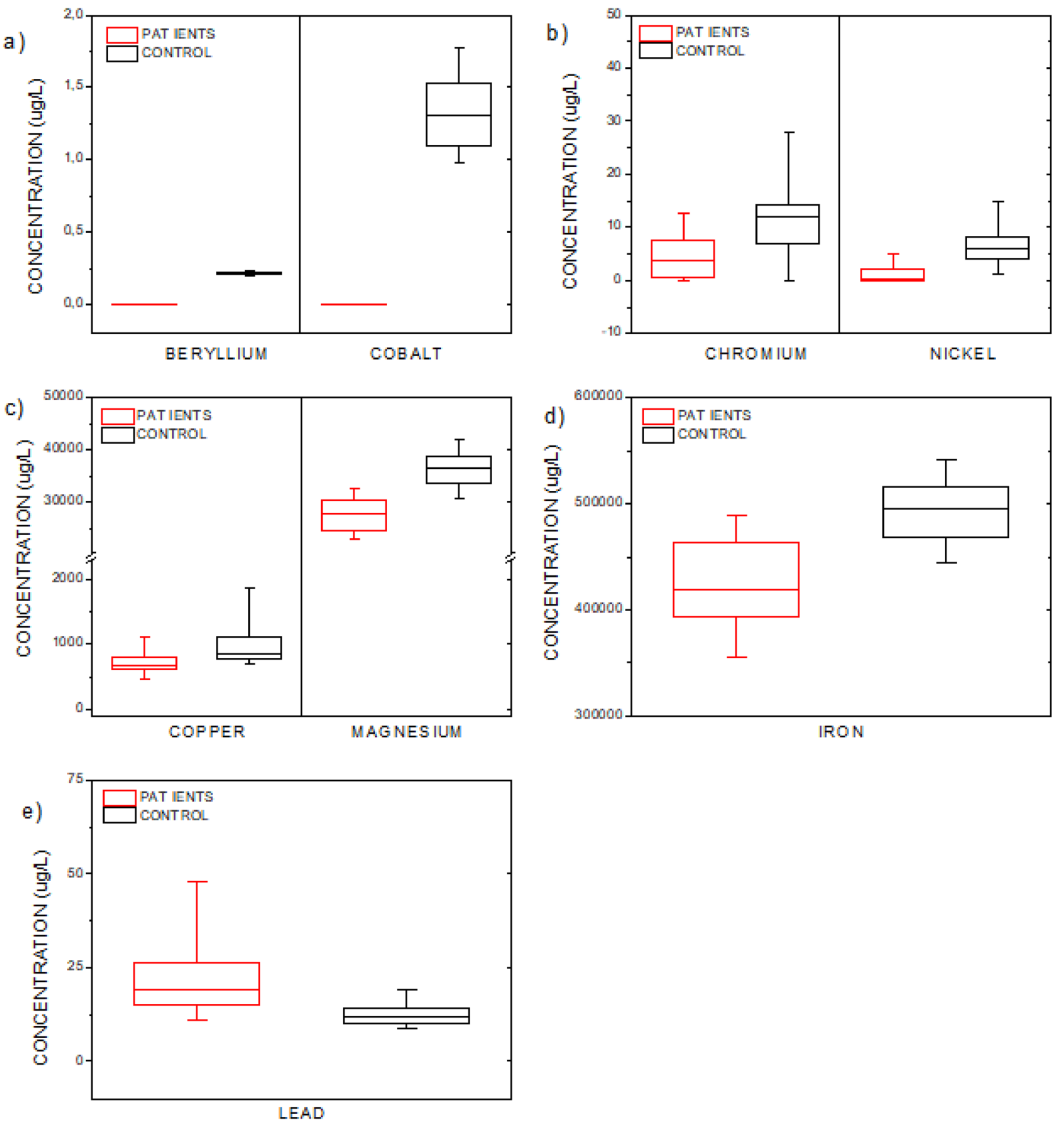
(**a**) Beryllium and cobalt; (**b**) Chromium and nickel; (**c**) Copper and magnesium; (**d**) Iron and (**e**) Lead concentration levels in patients with multiple sclerosis and control group.

Figure 1 shows box plots of elements with significant differences in concentration levels (Be, Co, Cr, Cu, Fe, Mg, Ni and Pb) between the patients and control group. These results are interesting and important, since abnormalities of transition metal metabolism may be increasingly important in the pathogenesis of neurodegenerative diseases^21^. The decrease of most elements in the blood of patients with multiple sclerosis may be directly related to both the etiology and the consequences of the disease.

Figure 1a shows the differences observed for beryllium and cobalt. We detect very low concentrations of Be and Co in the blood of patients. As one of the biological functions of cobalt is to support myelin formation, the reduction in its levels would imply the progression of myelin degeneration^17^. Thus, given the absence of this metallic element, as observed in our patient group, one cannot exclude its possible role in multiple sclerosis, since this is a disease characterized by the process of demyelination. However, in the control group this metallic element may be responsible for the integrity of the myelin sheath.

A variation in chromium and nickel levels has also been observed, see Fig. 1b, and this is related to biological modifications. In the human body, nickel is involved in enzymatic reactions with carbon monoxide dehydrogenase which oxidizes the CO and the hydrogenase converts the Ni into NiH_2_^23,24^. However, the role of Ni in neurodegenerative diseases still needs to be further studied. Chromium also is a metal that can be stored by the body and can cause cytotoxicity^25^. We observed lower Cr levels in the patients, but this finding cannot be sustained or simply explained by previous observations. Figure 1.c shows the differences observed for copper and magnesium, which are both metallic elements that can accumulate in the brain^26^. In relation to copper, this metal has been observed to attenuate experimental autoimmune encephalomyelitis, and this would suggest a role for Cu in the progression of multiple sclerosis^27^. This metallic element is used in the synthesis of myelin, thus the deficiency observed in our results may potentially cause myelinopathy^28^. Magnesium has a significant influence on the nervous system by reducing the excitability of nerve cells^29^. A deficit this metal, as shown in Fig. 1.c, may induce a dysfunction in lymphocytes or nerve cells^30^^,^ and the depletion we observed may be the cause of the etiology of multiple sclerosis^29^.

Iron is important for myelin production and is vital for normal neuronal metabolism^31^^,^ and this element is thought to promote oxidative damage in pathological states by catalyzing the formation of free radicals^32^. Exposure to this metallic element is initially through food consumption, and toxic levels of Fe accumulation are usually due to disrupted Fe homeostasis and metabolism in the brain^33,34^. Thus, changes in iron levels may be harmful and contribute to the pathogenesis of neurological diseases, including multiple sclerosis^32^. In this study, we observed a decrease in the concentration of Fe in patients with MS (Fig. 1d), with values that followed the decrease observed by Forte et al.^17^. These low blood values in patients may suggest iron accumulation in the brain which may be responsible for the demyelination process. Although cerebral iron accumulation in neurodegenerative disorders has been appreciated for a century, its mechanisms are still poorly understood^35^. With respect to Pb, this was the only element that showed an increase in concentration levels for MS patients, see Fig. 1e. Since the industrial revolution, lead has been extensively dispersed in the environment, and it has been observed that its toxicity contributes to a variety of important diseases such as neurological disorders^36^. Exposure to this metallic element is one of the environmental factors considered to play a role in the etiopathogenesis of multiple sclerosis^22^. In humans, oral ingestion and inhalation are the major routes of lead exposure, and the main target of Pb-induced toxicity is the nervous system^33^. In our results, even though the MS group presented with values higher than the control group, the levels found were within the limits of biological tolerance^37^. Thus, the highest Pb concentration levels in patients can suggest an association with inflammatory processes and the progression of disease.

The available studies provide evidence of the involvement of environmental and occupational exposures as co-factors for multiple sclerosis^17^. The environmental factors may include metals used in manufacturing, metals that contaminate ecosystems and metals present in arable soils, in air and in food. Metallic elements are neuroactive elements that affect different parts of the body, including the central nervous system^20^. The low concentration of metallic elements in the body play a crucial role in various metabolic events, the development of the nervous system and myelination of the nerve fibers^13^. The connection of these elements with multiple sclerosis, considered the most prevalent demyelinating brain disease, has drawn a huge interest^20^. Myelin sheaths are particularly susceptible to oxidative damage, and since trace elements can increase the production of free radicals it is conceivable that they may contribute to this demyelinating process^23,38^. ICP-MS analysis showed that mean concentrations of the studied metals were different in the blood of MS patients as compared with controls. The low blood values found may suggest the accumulation of these elements in the central nervous system and brain^33^. In particular, all these elements presented lower mean values in MS patients, with the exception for lead that was lower in healthy individuals. These results support the hypothesis that metallic elements in blood may be a discriminating factor for MS.

## Conclusions

Our results showed a decrease in the concentrations of beryllium, copper, chromium, cobalt, nickel, magnesium and iron, and an increased concentration of lead in MS patients. According to our data, a possible role for the imbalance in the concentrations of metallic elements can be proposed as possible co-factor in multiple sclerosis. Abnormalities in transition metals in biological matrices levels have been reported in several neurological diseases. These results are important as until now, in Brazil, there have been no studies addressing the levels of metallic elements in multiple sclerosis. However, further investigation in MS is required to clarify the correlation with metallic elements, and association with environmental agents.

## Data Availability

All data generated or analyzed during this study are included in this published article.
